# C1q nephropathy in adults is a form of focal segmental glomerulosclerosis in terms of clinical characteristics

**DOI:** 10.1371/journal.pone.0215217

**Published:** 2019-04-19

**Authors:** Kipyo Kim, Hyung-Eun Son, Ji-Young Ryu, Hajeong Lee, Seung Hyeok Han, Dong-Ryeol Ryu, Jin Ho Paik, Sejoong Kim, Ki Young Na, Dong-Wan Chae, Ho Jun Chin, Se Won Oh

**Affiliations:** 1 Department of Internal Medicine, Seoul National University Bundang Hospital, Seongnam, Republic of Korea; 2 Korean GlomeuloNephritis Study Group, Korean Society of Nephrology, Seoul, Republic of Korea; 3 Department of Internal Medicine, Seoul National University Hospital, Seoul, Republic of Korea; 4 Kidney Research Institute, Seoul National University College of Medicine, Seoul, Republic of Korea; 5 Department of Internal Medicine, Yonsei University College of Medicine, Seoul, Republic of Korea; 6 Department of Internal Medicine, School of Medicine, Ewha Woman's University, Seoul, Republic of Korea; 7 Department of Pathology, Seoul National University Bundang Hospital, Seongnam, Republic of Korea; 8 Department of Internal Medicine, Seoul National University College of Medicine, Seoul, Republic of Korea; 9 Department of Internal Medicine, Korea University College of Medicine and Korea University Anam Hospital, Seoul, Republic of Korea; Icahn School of Medicine at Mount Sinai, UNITED STATES

## Abstract

Although C1q nephropathy (C1qN) was introduced three decades ago, the clinical significance and renal outcomes of C1qN remain unclear. This study aimed to evaluate the clinical characteristics of C1qN, including renal outcomes, by performing a matched comparison within a multicenter cohort. We enrolled 6,413 adult patients who underwent kidney biopsy between January 2000 and January 2018 at three tertiary hospitals in Korea. We compared the clinical characteristics of 23 patients with C1qN with those of patients with focal segmental glomerulosclerosis (FSGS) or minimal change disease (MCD) who were matched by age, sex, diabetic status, and a period of biopsy. Histological and clinical parameters in patients with C1qN were also evaluated according to the different pathological phenotypes. For a mean follow-up period of 92 months, 4 patients with C1qN (17.4%) developed end-stage renal disease (ESRD). None of the matched patients with MCD had ESRD, but 7 (30.4%) of patients with FSGS progressed to ESRD, which was not different from that of C1qN patients (p = 0.491). Laboratory and pathological findings, except segmental glomerulosclerosis, were not notably different between FSGS and C1qN. The presence of segmental glomerulosclerosis, mesangial hypercellularity, and podocyte effacement did not affect both the short- and long-term renal outcomes in patients with C1qN. Our study showed that the renal outcomes of C1qN are comparable with those of FSGS, and not with MCD. Specific pathological findings, including segmental glomerulosclerosis in C1qN, were not associated with renal outcomes, which may suggest homogeneity in the clinical features of C1qN.

## Introduction

C1q nephropathy (C1qN) was first proposed by Jennette and Hipp in 1985, defined as a mesangial dominant or co-dominant deposition of C1q without evidence of systemic lupus erythematosus [1]. C1qN appears to be more common in children and young adults. The prevalence of C1qN is reported to be approximately 1.9–6.0% in unselected series of renal biopsies, but in some pediatric studies, it has been reported to be as high as 16.0% [2–4]. C1qN presented with diverse clinical manifestations such as nephrotic syndrome, mild proteinuria with or without hematuria, recurrent gross hematuria, nephritic syndrome, acute kidney injury, and rapidly progressive glomerulonephritis [5–9]. The histological findings of C1qN are also known to be heterogeneous. While some authors suggested that C1qN is within the spectrum of minimal change disease/focal segmental glomerulosclerosis (MCD/FSGS), C1qN is largely classified into the following two histological variants according to the light microscopic features: podocytopathy variant, including MCD and FSGS, and mesangial proliferative glomerulonephritis (GN) variant [4, 10]. Over the past 30 years, several studies regarding the clinical characteristics, treatment, and outcome of C1qN have been published. However, whether C1qN is a distinct clinicopathological entity or is variant of other glomerular diseases is still controversial. The clinical implication of the C1qN diagnosis also needs to be evaluated.

Previous studies often reported that C1qN has a clinically complicated and steroid-resistant course [11–13]. However, owing to the heterogeneity of clinical and histological features, the outcomes of C1qN has been reported inconsistently; the prognosis was relatively good in the pediatric studies that included many MCD cases, but poor in the studies that included more FSGS cases. In addition, most studies have small sample sizes and no control group and may be biased due to their single-center nature, such as clinical indications for kidney biopsy. Therefore, whether C1qN had a different prognosis from that of the typical idiopathic FSGS or MCD is not clear. The present study was a multicenter cohort study that compared the clinical and pathological findings and short- and long-term outcomes of C1qN with those of FSGS and MCD in patients matched for relevant variables. In addition, the clinical characteristics and renal prognosis of patients with C1qN were also analyzed according to the presence of diverse histological parameters such as segmental glomerulosclerosis, mesangial hypercellularity, and other characteristics.

## Methods

### Patients

We enrolled 6,315 adult candidates aged ≥18 years who had renal biopsy between January 2000 and January 2018 at three tertiary referral hospitals in Korea, namely Seoul National University Hospital, Seoul National University Bundang Hospital, and Korea University Anam Hospital. We defined C1qN on the basis of the criteria described by Jennette and Falk [14], which was adopted by another study [15] as follows: 1) presence of ≥2+ C1q in the mesangium on immunofluorescence (IF), 2) corresponding mesangial or para-mesangial electron dense deposits on electron microscopy (EM), and 3) lack of clinical and pathological evidence of systemic lupus erythematosus. FSGS and MCD were defined in a review article [16]. We enrolled patients who had C1qN and been followed up for >3 months and randomly selected patients with FSGS and MCD who were matched to the patients with C1qN with age, sex, a period of renal biopsy, and diabetes mellitus status. Renal biopsy involved the ultrasonography-guided percutaneous gun biopsy technique, and all biopsy specimens were initially evaluated by independent renal pathologists blinded to patients’ outcome in each hospital. Detailed histologic reports and biopsy slides of patients with C1qN were re-reviewed by a single experienced pathologist after enrollment. The clinical, biochemical, and prescription data at the time of biopsy and the last follow-up were queried into the electronic medical record with the primary keys of the patients’ identification number and the date of renal biopsy. The estimated glomerular filtration rate (eGFR) was calculated using the Chronic Kidney Disease Epidemiology Collaboration (CKD-EPI) equation [17]. Hypertension was defined as systolic blood pressure (SBP) of ≥140 mmHg, diastolic blood pressure (DBP) of ≥90 mmHg, or taking antihypertensive medication to control blood pressure. The final outcome of the study was end-stage renal disease (ESRD) until April 2018, which was gathered from the ESRD registry of the Korean Society of Nephrology [18]. We also compared the percent change and rate of decline in eGFR during 6 months after renal biopsy. We calculated the percent change in eGFR by [(eGFR at 6 months − eGFR at renal biopsy] × 100/eGFR at renal biopsy) and the rate of decline in eGFR by [(eGFR at 6 months–eGFR at renal biopsy) × 2], with the results expressed in ml/min/1.73 m^2^/year. This study was approved by the institutional review board (IRB) of Seoul National University Bundang Hospital (IRB approval No. B-1707/408-106). Written consent was waived by the IRB because of the retrospective nature of the study, and all data were fully anonymized before access by the researchers.

### Renal pathology

Methods of renal pathological evaluation are described elsewhere [19]. All biopsies were evaluated using appropriate standards for renal biopsy, including hematoxylin and eosin, periodic acid-Schiff, Masson trichrome, and periodic acid methenamine silver stains for light microscopy (LM), IF staining using antibodies against IgA, IgG, IgM, C3, C1q, and kappa and lambda light chains, and EM examination. We semi-quantitatively assessed the renal changes of glomerular size, mesangial matrix, mesangial cellularity, and interstitial fibrosis. Scores ranged from normal to severe (absence of the lesion, focal mild changes: <25% lesion present, focal moderate changes: 25–50% lesions present, and focal marked or diffuse changes: ≥50% lesions present). The lesions were grouped into normal (score 0), mild (1), moderate (2), or severe (3). We also assessed the arteriolar fibro-intimal thickening, grouped as presence or absence. Pathologists evaluated the IF staining to determine whether linear, granular, peripheral, and mesangial deposits were present in the glomerulus for the 5 items, which were reported semi-quantitatively as negative, trace, and 1–4 positive. We transformed the results numerically as follows: negative to 0 points, trace to 0.5 points, and 1–4 positive to 1–4 points. Electron-dense deposits on EM were also described as mild, moderate, and severe deposits.

### Statistical analysis

Data were expressed as mean ± standard deviation for continuous variables and percentage for categorical variables. Differences were analyzed using the Mann-Whitney U test or Kruskal-Wallis test for continuous variables and the chi-square or Fisher's exact test for categorical variables, depending on the number of subgroups. The Kaplan-Meier method was used for the survival curve, and the statistical significance was calculated using the log-rank test. For the multivariate Cox proportional hazards analysis, variables were chosen on the basis of P values of <0.05 in a univariate analysis, along with age and sex. The receiver-operating characteristic (ROC) was evaluated to predict ESRD events. We considered P values of <0.05 to be statistically significant. All the analyses were performed using SPSS statistics version 23 (IBM, USA).

## Results

### Characteristics of C1qN at renal biopsy

After excluding 378 patients with lupus nephritis definitely diagnosed on the basis of clinical and pathologic findings, we identified 28 patients with C1q positivity ≥ 2+ and corresponding electron dense deposits on EM. Of these 28 patients, 23 patients with C1qN followed up for more than 3 months and enrolled in our analysis. Data on blood pressures, serum creatinine level, urine protein-to-creatinine ratio (UPCR), LM findings, immunofluorescent staining, and EM findings, prescription of immunosuppressive (IS) medication during follow-up period, and ESRD events were collected for all the patients (Table 1). Table 2 shows the characteristics at renal biopsy of the patients with C1qN. The mean age of enrolled patients was 40.8 ± 15.2 years, 47.8% were male, and no patients had diabetes mellitus. Ten patients (43.0%) had hypertension, two (9.1%) had coronary artery disease, and one (4.5%) had cerebrovascular accident. The serum creatinine level, eGFR, and UPCR at renal biopsy were 1.25 ± 0.85 mg/dL, 81.7 ± 36.1 ml/min/1.73 m^2^, and 3.510 ± 4.461 g/g creatinine, respectively. LM findings showed segmental glomerulosclerosis in 11 patients (47.8%), global glomerulosclerosis in 16 patients (69.6%), and glomerular crescent in one patient (4.3%). Mesangial cellularity and mesangial matrix were normal to mildly increased, and 8 (34.8%) patients had mesangial hypercellularity without segmental glomerulosclerosis. Moderate to severe changes of tubular atrophy, interstitial fibrosis, or interstitial inflammattion were observed in 7 patients (30.4%). The IF staining of the mesangium showed ≥2+ intensity of C1q deposition in all the patients. The intensity of deposition in IgG, M, A, or C3 was normal to trace except in one patient with 3+ deposition of IgM. All the patients showed mild to severe deposition of electron-dense material in the mesangium. Mild to moderate podocyte effacement was observed in 9 patients (39.1%); and severe effacement of podocyte, in 14 patients (60.9%). Presence of segmental glomerulosclerosis was related to high SBP and low eGFR (S1 Table). However, pathological findings were not so different according to the presence of segmental glomerulosclerosis. Increased mesangial cellularity was related to higher eGFR (101.2 ± 21.3 ml/min/1.73 m^2^ vs 64.8 ± 38.2 ml/min/1.73 m^2^, p = 0.016, S2 Table) and lower percentage of segmental glomerulosclerosis (0.9% ±1.6% vs 8.0% ± 7.7%, p = 0.019) than normal cellularity. Other pathological findings such as global glomerulosclerosis, tubular atrophy, interstitial fibrosis, interstitial inflammation, and arteriolar fibrointimal thickening tended to be milder in the patients with increased mesangial cellularity, although the differences were not significant. When we grouped the patients according to the grade of podocyte effacement (moderate or more vs normal to mild), UPCR tended to be higher in the patients with higher grade of effacement (4.69 ± 1.54 g/g creatinine vs 1.68 ± 2.38 g/g creatinine, p = 0.072). Pathological findings were not different according to the grade of podocyte effacement (data not shown).

**Table 1:** Numbers of valid values of parameters in this study.

	C1qN		FSGS		MCD	
Parameters	Valid	Missing	Valid	Missing	Valid	Missing
Findings at renal biopsy						
Age	23	0	23	0	23	0
Gender	23	0	23	0	23	0
Diabetes mellitus	23	0	23	0	23	0
Hypertension	23	0	23	0	23	0
Coronary artery disease	22	1	21	2	19	4
Cerebrovascular disease	22	1	20	3	19	4
SBP	23	0	23	0	23	0
DBP	23	0	23	0	23	0
HBsAg	18	5	19	4	20	3
Anti-HCV antibody	19	4	16	7	18	5
Hemoglobin	23	0	23	0	23	0
Glucose	23	0	22	1	23	0
Cholesterol	23	0	22	1	23	0
Protein	23	0	21	2	23	0
Albumin	23	0	22	1	23	0
Creatinine	23	0	23	0	23	0
GFR	23	0	23	0	23	0
UPCR	23	0	23	0	23	0
Pathologic findings	23	0	23	0	23	0
GFR at 6 months after biopsy	23	0	20	3	20	3
Immunosuppressive medications	23	0	23	0	23	0

C1qN: C1q nephropathy, FSGS: focal segmental glomerulosclerosis, MCD: minimal change disease, SBP: systolic blood pressure, DBP: diastolic blood pressure, HBsAg: surface antigen of hepatitis B virus, anti-HCV antibody: antibody to hepatitis C virus, GFR: estimated glomerular filtration rate by CKD-EPI equation, UPCR: urine protein to creatinine ratio with a unit of g/g creatinine

**Table 2:** Clinical and pathologic features of C1q nephropathy.

No	Age	Sex	Clinical findings at biopsy				LM findings										IF findings on mesangium					EM findings				IS	Outcome	
			SBP	DBP	GFR	UPCR	Glm No	Cell.	Mes.	Crs	GS	SS	T	IB	II	V	IgG	IgM	IgA	C3	C1Q	Ms	En	Ep	Ef.		GFR change	ESRD
Absence of segmental glomerulosclerosis																												
1	45.9	F	91	47	111	8.902	50	1	0	0.0	2.0	0.0	0	0	0	0	0	0	0	0	2	1	0	0	3	p,ctx	-21.2	-
2	62.1	M	133	82	102	3.624	30	1	0	0.0	23.3	0.0	0	0	0	0	0	0	0	0	2	1	0	0	3	p,cni	-23.8	-
3	55.5	F	123	71	54	1.110	2	0	1	0.0	50.0	0.0	3	3	1	1	0	0	0.5	0.5	2	2	2	0	3	p,aza	-17.0	-
4	33.8	F	130	80	116	2.094	7	1	0	0.0	0.0	0.0	1	1	1	0	0.5	0.5	0	0	2	1	0	0	1	-	-5.7	-
5	48.9	F	110	70	87	2.900	33	1	1	0.0	0.0	0.0	1	1	1	0	0	0	0	0	2	2	0	0	3	p,mmf	17.5	-
6	55.2	F	103	66	103	18.527	67	1	0	0.0	0.0	0.0	1	1	1	1	0	0.5	0	0	2	1	0	0	3	p,cni	-29.9	-
7	28.8	F	110	60	107	0.872	35	0	0	0.0	0.0	0.0	1	1	1	0	0	0.5	0	0	2	1	0	0	2	p	-3.1	-
8	33.4	M	120	80	88	0.672	67	1	1	0.0	19.4	0.0	1	1	1	0	0.5	0.5	0.5	0	2	1	0	0	1	-	0.0	-
9	26.2	F	90	60	126	10.590	54	1	0	0.0	30.0	0.0	1	2	2	0	0.5	0.5	0	0	2	1	0	0	3	p,cni,ctx	-4.9	-
10	20.5	M	109	67	127	0.073	36	1	1	0.0	0.0	0.0	1	0	0	0	0	0	0	0.5	2	1	1	1	3	-	3.2	-
11	25.1	M	120	70	20	0.839	7	0	0	0.0	57.0	0.0	3	3	3	1	0	3	0	0	2	1	0	0	3	rtx	6.6	142
12	19.2	M	118	76	123	0.040	42	0	0	0.0	0.0	0.0	0	0	0	0	0.5	0.5	0.5	0.5	2	1	0	0	1	-	-12.0	-
Presence of segmental glomerulosclerosis																												
13	75.8	M	105	58	53	6.571	11	0	0	0.0	44.0	16.0	2	2	2	1	0.5	0	0	0.5	2	1	0	0	3	-	-15.8	-
14	40.6	F	144	89	21	7.692	38	0	0	0.0	79.0	13.0	3	3	3	1	0.5	0.5	0	0	2	1	0	0	2	p	-41.0	14
15	44.4	M	124	80	85	0.278	15	0	1	0.0	0.0	7.0	2	2	2	1	0.5	0	0	0	3	1	0	0	3	-	-14.8	-
16	38.7	M	128	64	25	0.572	11	0	0	0.0	9.1	9.1	1	1	1	0	0.5	0	0	0	2	1	0	0	3	p,cni	167.6	-
17	43.5	M	130	80	61	5.400	34	1	0	0.0	2.0	2.9	1	1	1	0	0.5	0.5	0	0	2	1	0	0	3	p,cni	-8.0	69
18	30.9	F	140	100	67	2.800	21	0	0	0.0	10.0	24.0	1	1	1	0	0	0	0.5	0	2	1	0	0	3	p	-18.3	-
19	60.1	F	126	76	28	2.180	182	0	0	1.6	70.9	15.0	1	3	2	1	2	0	0	0.5	3	2	0	0	2	p	-6.0	74
20	39.2	M	113	75	76	0.744	98	1	1	0.0	24.5	2.0	1	1	1	1	0	0	0	0	3	1	0	0	1	-	0.0	-
21	57.7	F	125	75	59	3.436	15	0	0	0.0	13.4	6.7	1	0	0	1	0	0.5	0	0	2	1	0	0	3	p	4.8	-
22	33.7	F	125	75	118	0.769	43	1	1	0.0	23.2	4.6	1	1	1	0	0	0	0	0	2	1	0	0	1	-	-11.1	-
23	18.8	M	110	70	124	0.049	18	0	0	0.0	5.6	5.6	1	0	0	0	0	0	0	0	2	3	0	0	1	-	-21.5	-

No: number of patients, SBP: systolic blood pressure in mmHg, DBP: diastolic blood pressure in mmHg, GFR: estimated glomerular filtration rate by CKD-EPI equation, UPCR: urine protein to creatinine ratio in g/g creatinine, nd: not done, Glm No.: number of glomeruli included in light microscopic examination, Cell: mesangial cellularity; 1-mild increased, 0-normal cellularity, Mes: mesangial matrix; 0-normal, 1-increased matrix, Crs: percent of glomeruli with crescent, GS: percent of glomeruli with global glomerulosclerosis, SS: percent of glomeruli with segmental glomerulosclerosis, T: grade of tubular atrophy; 0-none, 1-mild, 2-moderate, 3-severe, IB: grade of interstitial fibrosis; 0-none, 1-mild, 2-moderate, 3-severe, II: grade of interstitial inflammation; 0-none, 1-mild, 2-moderate, 3-severe, V: grade of arteriolar fibrointimal thickening; 0-abscent, 1-present, IF: positivity of immunofluorescent staining, EM: electron microscopy, Ms: deposit on mesangium;0-none, 1-mild, 2-moderate, 3-severe, En; deposit on subendothelial space;0-none, 1-mild, 2-moderate, 3-severe, Ep: deposit on subepithelia space;0-none, 1-mild, 2-moderate, 3-severe, IS: prescription of immunosuppressive agent after renal biopsy during follow-up period; p-prednisolone, cni-calcineurin inhibitor, ctx-oral cyclophosphamide, rtx-rituximab, GFR change: change of GFR during 6 months after renal biopsy (%), ESRD: duration (months) of follow-up period until incident end stage renal disease.

### Outcomes of C1qN

**Fig 1:**
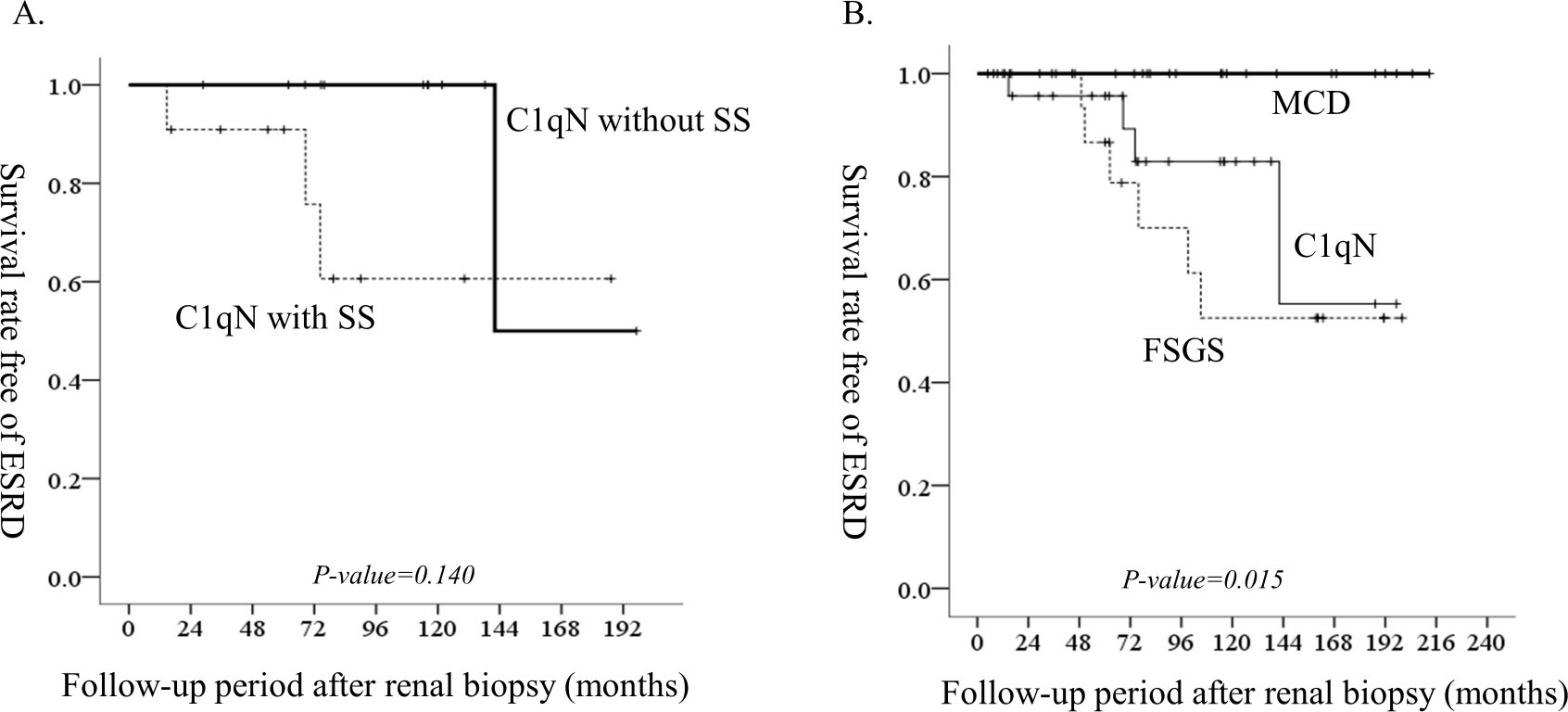
The incidence of end-stage renal disease. (A) Patients with C1qN according to the presence of segmental glomerulosclerosis (B) Patients with MCD, FSGS, and C1qN. ESRD: end-stage renal disease, MCD: minimal change disease, C1qN: C1q nephropathy, FSGS: focal segmental glomerulosclerosis, SS: segmental glomerulosclerosis. The P values were estimated using a log-rank test.

There was no mortality during the follow-up period. After renal biopsy, IS medications were prescribed in some patients (Table 2). Steroid was used in 13 patients (56.5%), and a calcineurin inhibitor was used in 5 (21.7%). IS treatment was not related to the percent change in eGFR and the change rate of eGFR during 6 months after biopsy. At 6 months after renal biopsy, the percent change in eGFR was −2.4% ± 39.3% and the change rate of eGFR was −12.2 ± 30.2 ml/min/1.73 m^2^/year as compared with eGFR at renal biopsy. The serum creatinine level at renal biopsy was the only factor related to the percent change in eGFR (Pearson correlation coefficient = 0.424, p = 0.044) and the change rate of eGFR (Pearson correlation coefficient = 0.493, p = 0.017) during 6 months after renal biopsy. Four patients (17.4%) developed ESRD during the follow-up period of 91.9 ± 47.9 months. The estimated 5- and 10-year ESRD-free survival rates were 95.7% and 82.8%, respectively. Presence of segmental glomerulosclerosis was not related to the development of ESRD (Fig 1), neither was mesangial hypercellularity or grade of podocyte effacement. eGFR was the most important factor to estimate ESRD (Table 3). eGFR of ≥60 ml/min/1.73 m^2^ at renal biopsy could estimate ESRD-free survival with a sensitivity of 78.9% and specificity of 100% by the ROC curve (p = 0.007, data not shown).

**Table 3:** Risk factors to incident ESRD among patients with C1q nephropathy.

	Model 1				Model 2				Model 3			
	HR	95% CI for HR		p-value	HR	95% CI for HR		p-value	HR	95% CI for HR		p-value
Age at biopsy (years)	uc	uc	uc	0.376	-	-	-	-	-	-	-	-
Gender (male)	uc	uc	uc	0.866	-	-	-	-	-	-	-	-
SBP (per 10 mmHg)	uc	uc	uc	0.458	-	-	-	-	-	-	-	-
GFR (per 10 ml/min/1.73 m^2^)	0.657	0.417	1.034	0.069	-	-	-	-	0.609	0.384	0.964	0.034
Global glomerulosclerosis (per 10%)	-	-	-	-	uc	uc	uc	0.776	-	-	-	-
Segmental glomerulosclerosis (present)	-	-	-	-	uc	uc	uc	0.371	-	-	-	-
Interstitial fibrosis (more than mild lesion)	-	-	-	-	uc	uc	uc	0.905	-	-	-	-
Interstitial inflammation (more than mild lesion)	-	-	-	-	9.225	0.946	89.955	0.056	uc	uc	uc	0.903
Tubular atrophy (present)	-	-	-	-	uc	uc	uc	0.804	-	-	-	-

SBP: systolic blood pressure, GFR: estimated glomerular filtration rate by CKD-EPI equation, uc: unable to calculate, HR: hazard ratio, Model 1: Cox’s hazard proportional model to estimate incident ESRD with clinical parameters, adjusted with age, gender, SBP and GFR at renal biopsy, Model 2: Cox’s hazard proportional model to estimate incident ESRD with pathologic parameters, adjusted with the findings of global glomerulosclerosis and segmental glomerulosclerosis, interstitial fibrosis, interstitial inflammation, and tubular atrophy, Model 3: Cox’s hazard proportional model to estimate incident ESRD adjusted with GFR and interstitial inflammation.

### C1qN, focal segmental glomerulosclerosis, and minimal change disease

We compared the clinical characteristics of the patients with FSGS or MCD with those of patients with C1qN, who were selected by matching age, sex, diabetes mellitus status, and period of biopsy-year (Table 4). The patients with C1qN had higher levels of serum protein and albumin, lower UPCR level, higher percentage of glomerulosclerosis, and higher interstitial fibrosis grade. However, the laboratory findings and LM findings of the patients with C1qN were not notably different from those of patients with FSGS, except the percentage of segmental glomerulosclerosis. The patients with C1qN had greater mesangial deposition of IgG (0.4 ± 0.6 vs 0.1 ± 0.2) and C1q (2.3 ± 0.3 vs 0.1 ± 0.3) and had more-intense mesangial electron-dense deposit as compared with the patients with FSGS (1.2 ± 0.4 vs 0.1 ± 0.3). Two patients with FSGS had trace staining of IgG in the mesangium, and only 2 patients with FSGS had mild electron-dense deposit in the mesangium. IS treatment was not markedly different between the patients with FSGS and those with C1qN. Any kind of IS medication was prescribed in 60.9% of patients with C1qN and in 43.5% of those with FSGS after renal biopsy (p = 0.238). No mortality occurred during the follow-up period in each group. There was no incident ESRD in the patients with MCD. However, the incidence rate of ESRD was 30.4% (7/23) in the patients with FSGS, which was not significantly different from that in the patients with C1qN (17.4%, p = 0.491). The estimated 5-year ESRD-free survival rate of the patients with FSGS was 83.7%, which was not significantly different from that of the patients with C1qN (Fig 1). When we predicted risk factors to estimate the prevalence of incident ESRD among C1qN and matched FSGS patients, eGFR at renal biopsy, the percentage of segmental glomerulosclerosis, and tubular atrophy grade were the independent risk factors (Table 5). eGFR of ≥70 ml/min/1.73 m^2^ at renal biopsy could estimate ESRD-free survival with a sensitivity of 71.4% and specificity of 100% on the basis of the ROC curve for C1qN and matched FSGS patients (AUC: 0.860, 95% confidence interval for AUC: 0.754–0.965, p < 0.001).

**Table 4:** Comparison of clinical characteristics among patients with C1qN, FSGS, and MCD.

	C1qN	FSGS	MCD	P-value
Number of patients	23	23	23	
Findings at renal biopsy				
Age (years)	40.8 ± 15.2	40.8 ± 15.3	41.5 ± 15.6	0.986
Gender (male, %)	47.8	47.8	47.8	1.000
Diabetes mellitus (%)	0.0	0.0	0.0	uc
Hypertension (%)	43.5	47.8	17.4	0.067
Coronary artery disease (%)	9.1	14.3	0.0	0.247
Cerebrovascular disease (%)	4.5	0.0	0.0	0.406
SBP (mmHg)	117.4 ± 13.0	129.4 ± 18.4	117.8 ± 10.9	0.078
DBP (mmHg)	72.7 ± 11.1	78.7 ± 11.6	71.0 ± 10.0	0.135
HBsAg (%)	4.6	0.0	0.0	0.332
Anti-HCV antibody (%)	5.3	0.0	5.6	0.637
Hemoglobin (g/dL)	13.9 ± 1.9	13.8 ± 2.1	13.8 ± 1.7	0.895
Glucose (mg/dL)	103.6 ± 23.1	102.2 ± 17.3	97.9 ± 20.5	0.555
Cholesterol (mg/dL)	243 ± 107	270 ± 129	347 ± 141	0.046
Protein (g/dL)	6.3 ± 1.3	6.1 ± 1.2	5.0 ± 1.5	0.007
Albumin (g/dL)	3.5 ± 1.0	3.6 ± 0.9	2.6 ± 1.0	0.004
Creatinine (mg/dL)	1.25 ± 0.85	1.41 ± 1.21	0.95 ± 0.28	0.380
GFR (ml/min/1.73 m2)	81.7 ± 36.1	77.0 ± 35.9	91.7 ± 25.1	0.289
UPCR (g/g creatinine)	3.510 ± 4.460	3.762 ± 4.127	8.119 ± 6.926	0.076
Renal pathologic findings by light microscopic examination				
Glomerular findings				
Number of glomeruli	39.8 ± 38.7	27.4 ± 16.7	30.2 ± 23.6	0.557
% of increased mesangial cellularity	47.8	47.8	30.4	0.386
% of global glomerulosclerosis	20.1 ± 24.3	24.5 ± 23.1	5.4 ± 7.8	0.006
% of segmental glomerulosclerosis	4.6 ± 6.7	8.0 ± 6.5	0.0 ± 0.0	<0.001
% of glomerular crescent	0.07 ± 0.33	0.22 ± 1.0	0.00 ± 0.00	0.602
% of increased mesangial matrix	30.4	39.1	8.7	0.053
Tubulointerstitial findings (%)				
Grade of interstitial fibrosis				0.013
none	26.1	4.3	43.5	
mild	43.5	52.2	52.2	
moderate	13.0	30.4	4.3	
severe	17.4	13.0	0.0	
Grade of interstitial inflammation				0.069
none	26.1	8.7	34.8	
mild	47.8	56.5	65.2	
moderate	17.4	26.1	0.0	
severe	8.7	8.7	0.0	
Grade of tubular atrophy				0.092
none	13.0	8.7	30.4	
mild	65.2	52.2	65.2	
moderate	8.7	21.7	4.3	
severe	13.0	17.4	0.0	
Vascular finding				
Presence of fibrointimal thickening (%)	39.1	34.8	17.4	0.238

C1qN: C1q nephropathy, FSGS: focal segmental glomerulosclerosis, MCD: minimal change disease, SBP: systolic blood pressure, DBP: diastolic blood pressure, HBsAg: surface antigen of hepatitis B virus, anti-HCV antibody: antibody to hepatitis C virus, GFR: estimated glomerular filtration rate by CKD-EPI equation, UPCR: urine protein to creatinine ratio with a unit of g/g creatinine, uc: unable to calculate

**Table 5:** Risk factors to incident ESRD among C1qN and matched FSGS patients.

	Model 1				Model 2				Model 3			
	HR	95% CI for HR		p-value	HR	95% CI for HR		p-value	HR	95% CI for HR		p-value
Age at biopsy (years)	uc	uc	uc	0.474	-	-	-	-	-	-	-	-
Gender (male)	uc	uc	uc	0.178	-	-	-	-	-	-	-	-
Pathologic diagnosis (FSGS)	uc	uc	uc	0.330	-	-	-	-	-	-	-	-
DBP (per 10 mmHg)	uc	uc	uc	0.194	-	-	-	-	-	-	-	-
SBP (per 10 mmHg)	1.800	1.121	2.892	0.015	-	-	-	-	uc	uc	uc	0.414
GFR (per 10 ml/min/1.73 m2)	0.681	0.531	0.874	0.003	-	-	-	-	0.757	0.595	0.963	0.023
Segmental glomerulosclerosis (%)	-	-	-	-	1.104	1.014	1.201	0.002	1.107	1.015	1.208	0.022
Global glomerulosclerosis (per 10%)	-	-	-	-	uc	uc	uc	0.089	-	-	-	-
Interstitial fibrosis (presence)	-	-	-	-	uc	uc	uc	0.254	-	-	-	-
Interstitial inflammation (presence)	-	-	-	-	uc	uc	uc	0.243	-	-	-	-
Tubular atrophy (present)	-	-	-	-	10.513	2.860	38.649	<0.001	5.831	1.358	25.044	0.018

SBP: systolic blood pressure, DBP: diastolic blood pressure, GFR: estimated glomerular filtration rate by CKD-EPI equation, uc: unable to calculate, HR: hazard ratio, Model 1: Cox’s hazard proportional model to estimate incident ESRD with clinical parameters, adjusted with age, gender, SBP, DBP, GFR, and pathologic diagnosis at renal biopsy, Model 2: Cox’s hazard proportional model to estimate incident ESRD with pathologic parameters, adjusted with the findings of global glomerulosclerosis and segmental glomerulosclerosis, interstitial fibrosis, interstitial inflammation, and tubular atrophy, Model 3: Cox’s hazard proportional model to estimate incident ESRD adjusted with GFR, segmental glomerulosclerosis, and tubular atrophy.

## Discussion

This study showed the short- and long-term renal prognosis in C1qN, which were not related to the presence of segmental glomerulosclerosis, mesangial hypercellularity, podocyte effacement grade, and other pathological parameters. The LM findings of C1qN were not markedly different from those of FSGS, except for the amount of segmental glomerulosclerosis. Furthermore, the renal prognosis in C1qN was also not significantly different from that in FSGS.

The overall outcome of C1qN has not been clearly determined yet and has been reported to be heterogeneous by histological phenotype. Therefore, most previous studies generally assessed the prognosis in C1qN separately in accordance with pathological variants. While the FSGS variant with nephrotic syndrome has a poor renal outcome, MCD and the mesangial proliferative GN variant seem to have a relatively good prognosis [10, 20, 21]. The total incidence of ESRD in our study was comparable to those of other studies of C1qN [10, 22], and there were 11 (47.8%) patients with segmental glomerulosclerosis, 11 (47.8%) with mesangial proliferation, 4 (17.4%) with no lesion in LM, and 14 (60.9%) with severe foot process effacement in EM. However, according to the presence of these pathological features, no significant differences were observed in other histological and clinical parameters except eGFR at renal biopsy and in renal outcome. This might suggest that the patients with C1qN in our study were roughly homogeneous in terms of clinical and histological characteristics. In addition, the increase in mesangial cellularity appeared to be associated with a higher baseline eGFR and lower degrees of segmental glomerulosclerosis and tubular atrophy. If C1qN progresses along the continuum between each pathological variant by the certain pathogenic mechanism, mesangial hypercellularity may be an initial change ahead of glomerulosclerosis and chronic tubulointerstitial lesion.

To date, the renal prognosis of C1qN has not been directly compared with those of other glomerular diseases in previous studies. In a study by Markowitz et al., 2 (18.2%) of 11 adult patients with C1qN presenting with a FSGS histology progressed to ESRD and showed 81.0 months of median renal survival [22]. The authors concluded that C1qN with a FSGS pattern has a renal outcome similar to that of idiopathic FSGS on the basis of data from the same institution. Meanwhile, our study clearly demonstrated that C1qN has a renal outcome comparable with that of FSGS by performing a matched comparison, which is consistent with the work of Markowitz et al. In age- and sex-matched C1qN and FSGS patients, the risk factors for ESRD did not include C1q positivity but only baseline GFR and chronic histological changes such as tubular atrophy and interstitial fibrosis. These findings may be helpful in making clinical decisions related to prognosis.

On the other hand, C1qN showed a renal outcome worse than that of MCD. C1qN studies related to MCD have been performed mainly in the pediatric population. Gunasekara et al. compared the outcome of patients with minimal change nephrotic syndrome with and without mesangial C1q deposition [12]. Patients with predominant C1q deposition showed a more frequent relapse but no significant difference in long-term renal outcome. However, the study of Gunasekara et al. was conducted in a very young pediatric population (median age, 4.5 years), and only the outcome of the MCD variant was evaluated. The outcome of C1qN appears to be associated with the age of the patients; pediatric patients have better prognosis and higher proportion of MCD histology than adult patients [13]. In our multivariate Cox regression models, tubular atrophy and segmental glomerulosclerosis are significantly associated with renal survival in C1qN and matched FSGS patients, but not in C1qN patients only. In previous literatures on primary FSGS, chronic tubulointerstitial lesions have been reported to affect renal survival [23, 24] and some of studies showed that glomerulosclerotic lesions also correlated with the development of ESRD [25], which may be reflected in our findings. Meanwhile, in patients with C1qN, these findings [26] may suggest relatively homogeneous renal outcome of C1qN variants or can be partly attributed to the small sample size.

The clinical significance and mechanism of C1q deposition in C1qN are still uncertain and only speculated. C1q is the subunit of C1, the first component in the activation of classical complement pathway [20]. Therefore, mesangial C1q deposition may be associated with complement activation by C1q binding to IgG within the immune complex. In early articles on C1qN, many cases of mesangial proliferative GN that support this mechanism were reported [2, 27–29]. However, some authors who consider C1qN as a spectrum of FSGS/MCD suggest that C1q deposits result from non-specific trapping associated with increased mesangial trafficking of plasma protein [22]. In our study, despite 8 patients with mesangial proliferation but not with segmental glomerulosclerosis, relatively lower staining intensities for immunoglobulins were observed compared with Vizjak et al.’s study. However, actually, the studies by Hisano et al. or Markowitz et al. showed lower mean fluorescence intensities for IgG and IgM, ranging from 0.2 to 1.3, which are not so different from our results. These differences between studies may result from two possible causes. First, it may reflect race differences between Asian and white populations. Our prevalence (0.35%) of C1qN is closest to those (0.4%) in Hisano et al.’s study, and specific pathologic features such as the degree of capillary wall deposits are also mostly similar to Hisano et al.’s. Second, in Vizjak et al.’s study, only 73.6% of total patients had electron microscopy examination, which can lead to inclusion of additional subjects who does not fully meet the diagnostic criteria including the presence of electron dense deposits in EM; the prevalence (1.9%) of C1qN in Vizjak et al.’s study was greater than that in Hisano et al.’s and Markowitz et al.’s (0.21%).

However, whether the C1q deposit in C1qN is the result of non-specific trapping, as in FSGS or MCD, is elusive. There are sporadic case reports that C1q deposition is associated with various clinical conditions and genetic factors, but the implications are still ambiguous [30–35]. If there is a shared mechanism in C1qN patients, the variants of C1qN can be integrated into a distinct disease entity; this can lead to the introduction and development of new therapeutic approaches. [36, 37]

Our findings may be somewhat limited by the small sample size and particularly by the low incidence of ESRD during the follow-up period. The homogeneity in the renal outcome of C1qN is not conclusive, which needs to be confirmed in larger studies. Nevertheless, the present study compared the renal outcome of patients with C1qN and matched patients with FSGS for the first time and has the advantage of a multicenter cohort study with long-term follow-up. In addition to segmental glomerulosclerosis, which has been discussed in most previous studies, the prognosis in C1qN was also evaluated on the basis of various pathological phenotypes such as increased mesangial cellularity and foot process effacement. Assessed renal outcomes encompassed both shot-term outcomes such as the change in eGFR over 6 months and long-term outcomes of ESRD.

In conclusion, our multicenter cohort study showed that the clinical features and short- and long-term renal outcome of C1qN are not notably different from those of FSGS, which further clarify the clinical features of C1qN in adult patients. Regardless of dominant C1q staining, adult C1qN present very similar features to FSGS in terms of clinical characteristics. In addition, the presence of segmental glomerulosclerosis and increased mesangial cellularity did not affect the prognosis in C1qN, which may suggest that C1qN is relatively homogeneous. Further studies are needed to identify the pathogenesis of C1qN and confirm the clinical course of each pathological variant.

## Supporting information

S1 Table Characteristics of C1qN according to the status of segmental glomerulosclerosis at renal biopsy. (DOCX)

S2 Table Characteristics of C1qN according to the status of mesangial cellularity at renal biopsy. (DOCX)

S1 File Supporting information file. (ZIP)
