# Supplementary material for: C1q nephropathy in adults is a form of focal segmental glomerulosclerosis in terms of clinical characteristics
Source: PLoS One. 2019 Apr 19;14(4):e0215217. doi: 10.1371/journal.pone.0215217 (PMC6474651; doi:10.1371/journal.pone.0215217)
Supplement: S1 Table — (DOCX) [file pone.0215217.s001.docx]

**S1 Table. Characteristics of C1qN according to the status of segmental sclerosis on glomeruli at renal biopsy**

|  | Absence | Presence | P-value |
| --- | --- | --- | --- |
| Number of patients | 12 | 11 |  |
| Findings at renal biopsy |  |  |  |
| Age (years) | 38.9 ± 14.9 | 43.9 ± 15.6 | 0.413 |
| Gender (male, %) | 41.7 | 54.5 | 0.537 |
| Diabetes mellitus (%) | 0.0 | 0.0 | uc |
| Hypertension (%) | 41.7 | 45.5 | 0.855 |
| Coronary artery disease (%) | 0.0 | 18.2 | 0.476 |
| Cerebrovascular disease (%) | 0.0 | 9.1 | 1.000 |
| SBP (mmHg) | 113.1 ± 13.7 | 124.6 ± 11.9 | 0.044 |
| DBP (mmHg) | 69.1 ± 10.1 | 76.6 ± 11.3 | 0.211 |
| HBsAg (%) | 10.0 | 0.0 | 1.000 |
| Anti-HCV antibody (%) | 10.0 | 0.0 | 1.000 |
| Hemoglobin (g/dL) | 14.1 ± 1.7 | 13.7 ± 2.2 | 0.786 |
| Glucose (mg/dL) | 100.4 ± 19.8 | 107.1 ± 26.8 | 0.833 |
| Cholesterol (mg/dL) | 273 ± 128 | 209 ± 70 | 0.566 |
| Protein (g/dL) | 6.2 ± 1.5 | 6.4 ± 1.0 | 0.880 |
| Albumin (g/dL) | 3.3 ± 1.2 | 3.7 ± 0.6 | 0.487 |
| Creatinine (mg/dL) | 1.05 ± 0.92 | 1.48 ± 0.75 | 0.016 |
| GFR (ml/min/1.73 m2) | 96.9 ± 31.8 | 65.0 ± 34.3 | 0.044 |
| UPCR (g/g creatinine) | 4.19 ± 5.67 | 2.77 ± 2.70 | 0.740 |
| Renal pathologic findings in light microscopic examination | | | |
| Glomerular findings |  |  |  |
| Number of glomeruli | 35.8 ± 22.1 | 44.2 ± 52.1 | 0.833 |
| % of increased mesangial cellularity | 66.7 | 27.3 | 0.100 |
| % of global glomerulosclerosis | 15.1 ± 20.9 | 25.6 ± 27.4 | 0.169 |
| % of segmental glomerulosclerosis | 0.0 ± 0.0 | 9.6 ± 6.7 | <0.001 |
| % of glomerular crescent | 0.00 ± 0.00 | 0.15 ± 0.48 | 0.740 |
| % of increased mesangial matrix | 33.3 | 27.3 | 1.000 |
| Tubulointerstitial findings (%) |  |  |  |
| Grade of interstitial fibrosis |  |  | 0.811 |
| none | 33.3 | 18.2 |  |
| mild | 41.7 | 45.5 |  |
| moderate | 8.3 | 18.2 |  |
| severe | 16.7 | 18.2 |  |
|  |  |  |  |
| Grade of interstitial inflammation |  |  | 0.633 |
| none | 33.3 | 18.2 |  |
| mild | 50.0 | 45.5 |  |
| moderate | 8.3 | 27.3 |  |
| severe | 8.3 | 9.1 |  |
| Grade of tubular atrophy |  |  | 0.147 |
| none | 25.0 | 0.0 |  |
| mild | 58.3 | 72.7 |  |
| moderate | 0.0 | 18.2 |  |
| severe | 16.7 | 9.1 |  |
| Vascular finding |  |  |  |
| Presence of fibrointimal thickening (%) | 25.0 | 54.5 | 0.214 |

C1qN: C1q nephropathy, SBP: systolic blood pressure, DBP: diastolic blood pressure, HBsAg : surface antigen of hepatitis B virus, anti-HCV antibody: antibody to hepatitis C virus, GFR: estimated glomerular filtration rate by CKD-EPI equation, UPCR: urine protein to creatinine ratio with a unit of g/g creatinine, uc: unable to calculate
