# Supplementary material for: C1q nephropathy in adults is a form of focal segmental glomerulosclerosis in terms of clinical characteristics
Source: PLoS One. 2019 Apr 19;14(4):e0215217. doi: 10.1371/journal.pone.0215217 (PMC6474651; doi:10.1371/journal.pone.0215217)
Supplement: S2 Table — (DOCX) [file pone.0215217.s002.docx]

**S2 Table. Characteristics of C1qN according to the status of cellularity in glomeruli at renal biopsy**

|  | Normal | Increased | P-value |
| --- | --- | --- | --- |
| Number of patients | 12 | 11 |  |
| Findings at renal biopsy |  |  |  |
| Age (years) | 42.3 ± 18.0 | 40.2 ± 12.4 | 0.924 |
| Gender (male, %) | 50.0 | 45.5 | 0.827 |
| Diabetes mellitus (%) | 0.0 | 0.0 | uc |
| Hypertension (%) | 58.3 | 27.3 | 0.214 |
| Coronary artery disease (%) | 16.7 | 0.0 | 0.481 |
| Cerebrovascular disease (%) | 8.3 | 0.0 | 1.000 |
| SBP (mmHg) | 122.7 ± 11.5 | 114.0 ± 15.2 | 0.288 |
| DBP (mmHg) | 74.1 ± 11.8 | 71.1 ± 10.7 | 0.880 |
| HBsAg (%) | 0.0 | 12.5 | 0.444 |
| Anti-HCV antibody (%) | 0.0 | 11.1 | 0.474 |
| Hemoglobin (g/dL) | 13.3 ± 1.9 | 14.6 ± 1.7 | 0.069 |
| Glucose (mg/dL) | 100.9 ± 25.0 | 97.4 ± 20.2 | 0.211 |
| Cholesterol (mg/dL) | 208 ± 58 | 280 ± 136 | 0.211 |
| Protein (g/dL) | 6.5 ± 1.0 | 6.1 ± 1.5 | 0.651 |
| Albumin (g/dL) | 3.8 ± 0.7 | 3.2 ± 1.2 | 0.413 |
| Creatinine (mg/dL) | 1.64 ± 1.02 | 0.82 ± 0.28 | 0.006 |
| GFR (ml/min/1.73 m2) | 64.8 ± 38.2 | 101.2 ± 21.3 | 0.016 |
| UPCR (g/g creatinine) | 2.20 ± 2.55 | 4.94 ± 5.69 | 0.235 |
| Renal pathologic findings in light microscopic examination | | | |
| Glomerular findings |  |  |  |
| Number of glomeruli |  |  |  |
| % of global glomerulosclerosis | 28.3 ± 29.8 | 11.3 ± 12.4 | 0.260 |
| % of segmental glomerulosclerosis | 8.03 ± 7.74 | 0.86 ± 1.59 | 0.019 |
| % of glomerular crescent | 0.13 ± 0.46 | 0.00 ± 0.00 | 0.740 |
| % of increased mesangial matrix | 16.7 | 45.5 | 0.193 |
| Tubulointerstitial findings (%) |  |  |  |
| Grade of interstitial fibrosis |  |  | 0.117 |
| none | 25.0 | 27.3 |  |
| mild | 25.0 | 63.6 |  |
| moderate | 16.7 | 9.1 |  |
| severe | 33.3 | 0.0 |  |
|  |  |  |  |
| Grade of interstitial inflammation |  |  | 0.286 |
| none | 25.0 | 27.3 |  |
| mild | 33.3 | 63.6 |  |
| moderate | 25.0 | 9.1 |  |
| severe | 16.7 | 0.0 |  |
| Grade of tubular atrophy |  |  | 0.117 |
| none | 8.3 | 18.2 |  |
| mild | 50.0 | 81.8 |  |
| moderate | 16.7 | 0.0 |  |
| severe | 25.0 | 0.0 |  |
| Vascular finding |  |  |  |
| Presence of fibrointimal thickening (%) | 58.3 | 18.2 | 0.089 |

Increased: mild increase of cellularity in glomeruli observed by light microscopy, C1qN: C1q nephropathy, SBP: systolic blood pressure, DBP: diastolic blood pressure, HBsAg : surface antigen of hepatitis B virus, anti-HCV antibody: antibody to hepatitis C virus, GFR: estimated glomerular filtration rate by CKD-EPI equation, UPCR: urine protein to creatinine ratio with a unit of g/g creatinine, uc: unable to calculate
